# Intranasal delivery of a bivalent norovirus vaccine formulated in an *in situ* gelling dry powder

**DOI:** 10.1371/journal.pone.0177310

**Published:** 2017-05-18

**Authors:** Jordan P. Ball, Michael J. Springer, Yawei Ni, Isaac Finger-Baker, Juan Martinez, Jessica Hahn, John F. Suber, Ashley V. DiMarco, James D. Talton, Ronald R. Cobb

**Affiliations:** Research and Development Department, Nanotherapeutics, Inc., Alachua, Florida, United States of America; University of Minnesota College of Veterinary Medicine, UNITED STATES

## Abstract

The global health community is beginning to understand the burden of norovirus-associated disease, which has a significant impact in both developed and developing countries. Norovirus virus like particle (VLP)-based vaccines are currently under development and have been shown to elicit systemic and mucosal immune responses when delivered intranasally. In the present study, we describe the use of a dry powder formulation (GelVac^™^) with an *in situ* gelling polysaccharide (GelSite^™^) extracted from *Aloe vera* for nasal delivery of a bivalent vaccine formulation containing both GI and GII.4 norovirus VLPs. Dose-ranging studies were performed to identify the optimal antigen dosages based on systemic and mucosal immune responses in guinea pigs and determine any antigenic interference. A dose-dependent increase in systemic and mucosal immunogenicity against each of the VLPs were observed as well as a boosting effect for each VLP after the second dosing. A total antigen dose of ≥50 μg of each GI and GII.4 VLPs was determined to be the maximally immunogenic dose in guinea pigs. The immunogenicity results of this bivalent formulation, taken together with previous work on monovalent GelVac^™^ norovirus vaccine formulation, provides a basis for future development of this norovirus VLP vaccine.

## Introduction

Viruses belonging to the genus *Norovirus* are responsible for over 90% of all non-bacterial gastroenteritis epidemics and are a leading cause of global diarrhea [1–7]. Norovirus accounts for approximately 23 million infections per year [4] and approximately 220,000 deaths in developing countries [8, 9]. Although norovirus affects all ages, children younger than 5 years [5, 10], the elderly [11], and immunocompromised persons [12] are at greatest risk. There is a need for a prophylactic norovirus vaccine and promising virus-like particle (VLP)-derived vaccine candidates are under development [13–19]. The genetic and antigenic diversity of noroviruses are a challenge to vaccine development with more than 30 genotypes within genogroups I (GI) and II (GII) [20–22] known to infect humans. Due to this wide variation, development of a broadly effective vaccine remains a challenge as the antibodies from humans immunized against one genogroup do not cross react with noroviruses from other genogroups [23]. Recently, genogroup II has become the most prevalent, accounting for 81.4% of norovirus outbreaks worldwide [24]. Several recently described cell culture systems for human norovirus have not been replicated or have only shown limited viral replication [25–28]. However, a recent report demonstrates that viral replication was established in human intestinal epithelial cells [29]. This new cell culture system will allow the development of new methods to assess the efficacy of new treatment regimens for norovirus infections. To date, these systems have not been developed sufficiently to support large scale viral production. Norovirus VLPs, either expressed in plants or insect cells which are structurally and morphologically similar to the native virus, are widely used as a source of norovirus antigen [14, 15, 18, 19, 30, 31].

Establishment of a clear immune correlate of protection has been an obstacle toward the development of such vaccine candidates. Research has shown that histo-blood group antigens (HBGAs) serve as attachment factors or receptors for noroviruses in a genotype specific manner [13, 32, 33]. In addition, the lack of HBGAs were shown to restrict viral replication [29]. Histo-blood group antigens are complex carbohydrates expressed on red blood cells and, in individuals with a functional fucosyltransferase 2 (*FUT 2*) gene, also on mucosal surfaces and body secretions [34]. An established correlate of protection to norovirus infection is the ability of serum and mucosal antibodies to block binding of norovirus VLPs to HBGAs [31, 35, 36]. Norovirus specific serum IgGs alone were not protective in challenge models in humans [35, 37, 38]. In contrast, norovirus specific serum IgA and salivary IgA levels have been associated with protection against norovirus gastroenteritis [35, 39, 40]. Previous studies have shown different B- and T-cell responses are observed in mice immunized intranasally and intramuscularly with norovirus VLPs [41]. Recent studies have shown that mucosal IgA antibodies are induced by intranasal administration of norovirus VLPs and not by intramuscular administration [36].

Previous studies have shown that the incorporation of VLPs with GelVac^™^ nasal dry powder formulation is able to increase antigen availability and induce systemic immunity as well as both local and distal mucosal immunity [16, 19]. Intranasal immunization of guinea pigs with the GelVac^™^ norovirus vaccine resulted in high levels of mucosal IgA and serum IgG antibodies in a dose-dependent manner [14, 16]. The present study investigating a bivalent norovirus vaccine extends the previous work with monovalent vaccine formulations [14, 16] and demonstrates that a GelVac^™^ formulated bivalent norovirus GI/GII.4 VLP vaccine induces high levels of antigen-specific systemic and mucosal antibodies in a dose-dependent manner to both GI and GII.4 VLPs. The bivalent GelVac^™^ norovirus vaccine formulation also induced neutralizing antibodies to both GI and GII.4 VLPs in a similar fashion as that observed in previous studies [14]. The results presented in the present study form the basis for future studies to investigate a bivalent GelVac^™^ GI/GII.4 norovirus vaccine formulation for the prevention of norovirus-induced gastroenteritis in humans.

## Materials and methods

### GI and GII.4 vaccine formulation

Recombinant norovirus GI (8K/1979/USA) and GII.4 (Minerva/2006/USA) VLPs, consisting solely of the VP1 major capsid protein, were expressed in *Spodoptera frugiperda* (Sf9) using the baculovirus expression system (Invitrogen Life Technologies, Carlsbad, CA). Sf9 cells were cultured at the 1 L scale and infected with baculovirus clones encoding for GI or GII.4 VLPs at a multiplicity of infection of 0.1 at a cell density above 1x10^6^ cells/mL. The infected cells were cultured until the viable cell density was <30% (approximately 4–5 days). Primary clarification was performed by Millistak+ HC μPod D0HC depth filtration media (23 cm^2^), (Millipore, Billerica, MA) followed by microfiltration through an Opticap XL150 Millipore Express SHC (0.5/0.2 μm) filter (Millipore, Billerica, MA). Clarified supernatant was diluted 3-fold with 50 mM sodium acetate, pH 5.0 for cation exchange chromatography.

Cation exchange chromatography was performed on POROS HS50 resin using an Akta Avant 150 chromatography skid (GE Life Sciences, Marlborough, MA). After equilibration with 20 mM sodium acetate buffer, pH 5.0, clarified supernatant was loaded at 300 cm/hr, washed with 20 mM sodium acetate buffer and eluted with a linear gradient of NaCl (0 to 1 M NaCl). Purified drug substance was ultrafiltered using a 100 kDa Vivaflow 50 and diafiltered with Mg^2+^, Ca^2+^-free PBS (Sartorius, Bohemia, NY). Samples were analyzed by SDS-PAGE, western blot, and ELISA for GI/GII.4 norovirus VLP content.

The size of the stock VLP particles was determined using transmission electron microscopy at University of Florida, Interdisciplinary Center for Biotechnology Research, Electron Microscopy and Bio-imaging Core. VLP stocks were diluted in water for imaging. Carbon coated Formvar copper grids, 400 mesh were glow discharged with Pelco easiGlow unit. Samples were incubated on the grids for 5 minutes. Excess solution was removed with filter paper. The sample grid was then incubated with 1% aqueous uranyl acetate 30 seconds. Excess stain was removed with filter paper, air dried, and examined with a Hitachi H-7000 TEM (Hitachi High Technologies America, Inc. Schaumburg, IL) and digital images acquired with a Veleta 2k x 2k camera and iTEM software (Olympus Soft-Imaging Solutions Corp, Lakewood, CO).

The particle size distribution for each of the powders was performed by using a 50 mg sample of each powder and suspending it in 100% isopropanol. Particle size distribution, by volume, was determined using a laser diffraction particle size analyzer with a liquid module (Beckman Coulter LS13-320, Pasadena, CA). Performance of the instrument was verified using a 35 μm garnet reference standard.

The GelVac^™^ vaccine powders were produced using a lyophilization-milling method. Norovirus VLPs were formulated in liquid using a proprietary formulation in GelSite^®^ polymer and then lyophilized. The dried formulation contained 0.25% (w/w) GelSite^®^ polymer and various amounts of VLP (based on BCA and ELISA data). Placebo control powders were formulated in a similar manner but did not contain VLPs. Each individual formulation was milled using a mortar and pestle in a controlled environment (< 10% relative humidity) and passed through a 70 μM filter. The doses were stored in sealed containers room temperature under desiccation prior to use. The non-adjuvanted GelVac^™^ vaccine dry powders were produced in sufficient quantity for the entire study and characterized using ELISA, SDS-PAGE and western blot data as previously described and shown to be greater than 95% pure [14]. Final dosages were calculated based on ELISA data as previously described [14].

### Animal studies

The animal protocols used in this study were approved by the Institutional Animal Care and Use Committee at the University of Florida. The Association for Assessment and Accreditation of Laboratory Animal Care (AAALAC) International recommendations were followed for general procedures with respect to animal care and housing. Female Hartley guinea pigs (Harlan Laboratories), six to eight weeks of age and 200–250 g were utilized for these studies. Animals were allowed to acclimate for two weeks prior to study. Animals were fed commercial guinea pig food and Timothy hay once per day and water was provided *ad libitum*. Animals were not fasted overnight prior to each administration of vaccine. Animals were monitored daily and weighed twice weekly throughout the study. Female (350 g) Hartley guinea pigs obtained from Harlan Laboratories were selected as the model animal for evaluation of the intranasal dry power delivery. Varying concentrations of a GelVac^™^ dry powder vaccine containing both GI and GII.4 VLPs were used to observe the dose response of guinea pigs (Table 1).

**Table 1:** Animal study experimental design.

Group	Formulation	rVLP Dose (μg)#	Antigen Presentation Schedule (Study Day)	Antigen Administration Route (Respective)	Sample Collection Schedule (Study Day)	Group Size (n)
1	Control	0	0, 21	IN, IN	0, 7, 14, 21, 42, 56	4
2	GI & GII Antigen	5	0, 21	IN, IN	0, 7, 14, 21, 42, 56	4
3	GI & GII Antigen	15	0, 21	IN, IN	0, 7, 14, 21, 42, 56	4
4	GI & GII Antigen	50	0, 21	IN, IN	0, 7, 14, 21, 42, 56	4
5	GI & GII Antigen	100	0, 21	IN, IN	0, 7, 14, 21, 42, 56	4
6	GI Antigen	50	0, 21	IN, IN	0, 7, 14, 21, 42, 56	4
7	GII Antigen	50	0, 21	IN, IN	0, 7, 14, 21, 42, 56	4

^#^Amount of each VLP included in each dose. In Group 4, 5μg of each VLP was added for a total of 10 μg of VLPs were added in the total dose.

Animals were randomly distributed into study groups (n = 4) and allowed to acclimate for two weeks, prior to immunization. Each study consisted of seven study groups, one for placebo powders and four for GelVac^™^ VLP bivalent dry powder vaccines at different antigen dose levels and two monovalent vaccine formulations. Each animal group received an enumerated dose of antigens suspended in GelVac^™^ deposition powder on an administration day. Powder vaccines were administered intranasally on days 0 and 21 with 10 mg/nare of dry powder formulation. Each bivalent dose and monovalent controls had a total amount of each antigen as described in Table 1 (e.g. in Group 4, 5 μg GI and 5 μg GII.4 antigens in the total administration dose). The control dosage was based upon previous data [14, 16]. Aptar Unit Dose Spray (UDS) Devices (Aptar Pharma, Congers, NY) equipped with small animal adapters were each loaded with 10 mg of vaccine powder were used for intranasal powder delivery. Delivery of greater than 92% of the powder has been demonstrated using these devices (data not shown). Guinea pigs were anesthetized with 5% isoflurane prior to immunization and sample collection. While under anesthesia, each animal was given two administrations of VLP powders, one per nare with half of the total antigen dose per nare (10 mg total powder per nare). A placebo powder formulation was administered similarly to the control group.

Guinea pig serum and vaginal lavage samples were collected on days 0 (preimmunization), 21 (preimmunization), 42, and 56. Blood, (1 mL/collection period) was collected from the superior vena cava and allowed to coagulate in serum separation tubes. Serum was collected as the supernatant after centrifugation for 10 min at 6000 rpm. Vaginal lavages were collected by lavaging 300 μL of PBS for 60 s in the vaginal tract with an oral feeding tube. Gastrointestinal samples were obtained on day 56. On day 56, guinea pigs were maintained under 5% isoflurane and exsanguinated by cardiocentesis. Gastrointestinal samples were collected by lavaging the 12 cm of the small intestines by passing 1 mL of PBS through the area three times. All samples were clarified by centrifugation for 10 min at 6000 rpm and stored at -20°C prior to analysis.

### ELISA for serum and mucosal antibodies

ELISA for serum and mucosal antibodies were performed as previously described [14]. Briefly, VLPs in PBS were incubated on Nunc MaxiSorp 96-well plates (Fisher Scientific) for 4 h at room temperature. The plates were blocked overnight at 4°C. All samples were serially diluted 2-fold and allowed to incubate at room temperature for 1 h. Plates were washed 5 times, followed by incubation with anti-guinea pig IgG-HRP (Southern Biotech, Birmingham, AL at 1:1000, anti-guinea-pig IgA (Creative Diagnostics, Shirley, NY) at 1:1000, anti-guinea pig IgG1 HRP (Antibodies Online) at 1:1000, or anti-guinea pig IgG2 RP (Antibodies Online) at 1:2000 for 1 h at room temperature. The plates were washed 5 times and developed with 1-step Ultra TMB according to the manufacturer’s protocol. End-point titers were reported as the reciprocal of the highest dilution that produced an OD of 0.1 above background and presented as Geometric Mean Titers. ositive control serum generated in guinea pigs against GI or GII.4 was included in each test run to confirm reproducibility.

### Gastric mucin ligand-binding serum neutralization assay

Porcine Gastric Mucin Type III (PGM) (Sigma Aldrich) has been previously used as a substrate for norovirus VLP antibody-blockade assays [42, 43]. Gastric mucin ligand-binding serum neutralization assays were performed as described. [14]. Briefly, PGM was dissolved in PBS at a final concentration of 5 mg/mL and 100 μL was added to each well of a 96-well MaxiSorp plate and incubated 4 h at room temperature. The wells were blocked overnight at 4°C. Serum samples were serially diluted and norovirus GI or GII.4 VLPs were added to the hyperimmune serum (0.25 μg/mL) and incubated for 1 h at room temperature. A total of 100 μL of the VLP-hyperimmune serum was added to each well and incubated for 1 h at room temperature. The wells were washed 5 times followed by the addition of goat-anti-mouse-HRP conjugate antibody (Milllipore, 1:2000 dilution) and incubated 1 h at room temperature. Plates were developed using 1-step Ultra TMB substrate according to the manufacturer’s protocol. Each serum sample was tested in 2-fold serial dilution and neutralizing titers were reported as the inflection of the curve (50% reduction, EC50) as indicated by the 4-Parameter Logistics fit.

### Statistics

SoftMax Pro 4.8 was used for sigmoidal curve fits. Two-way analysis of variance (ANOVA) models were fit separately to each sample type (serum, neutralizing, and vaginal lavage) (GraphPad Prism 7.01 Software, La Jolla, CA). Each titer was transformed by taking the base-10 logarithm of the measurement values. Tukey’s post-hoc test for multiple comparisons was used to compare each dose group (GraphPad Prism 7.01 Software). All results are reported based on the p ≤ 0.05 level of significance.

## Results

### GelVac^™^ vaccine powder characterization

**Fig 1:**
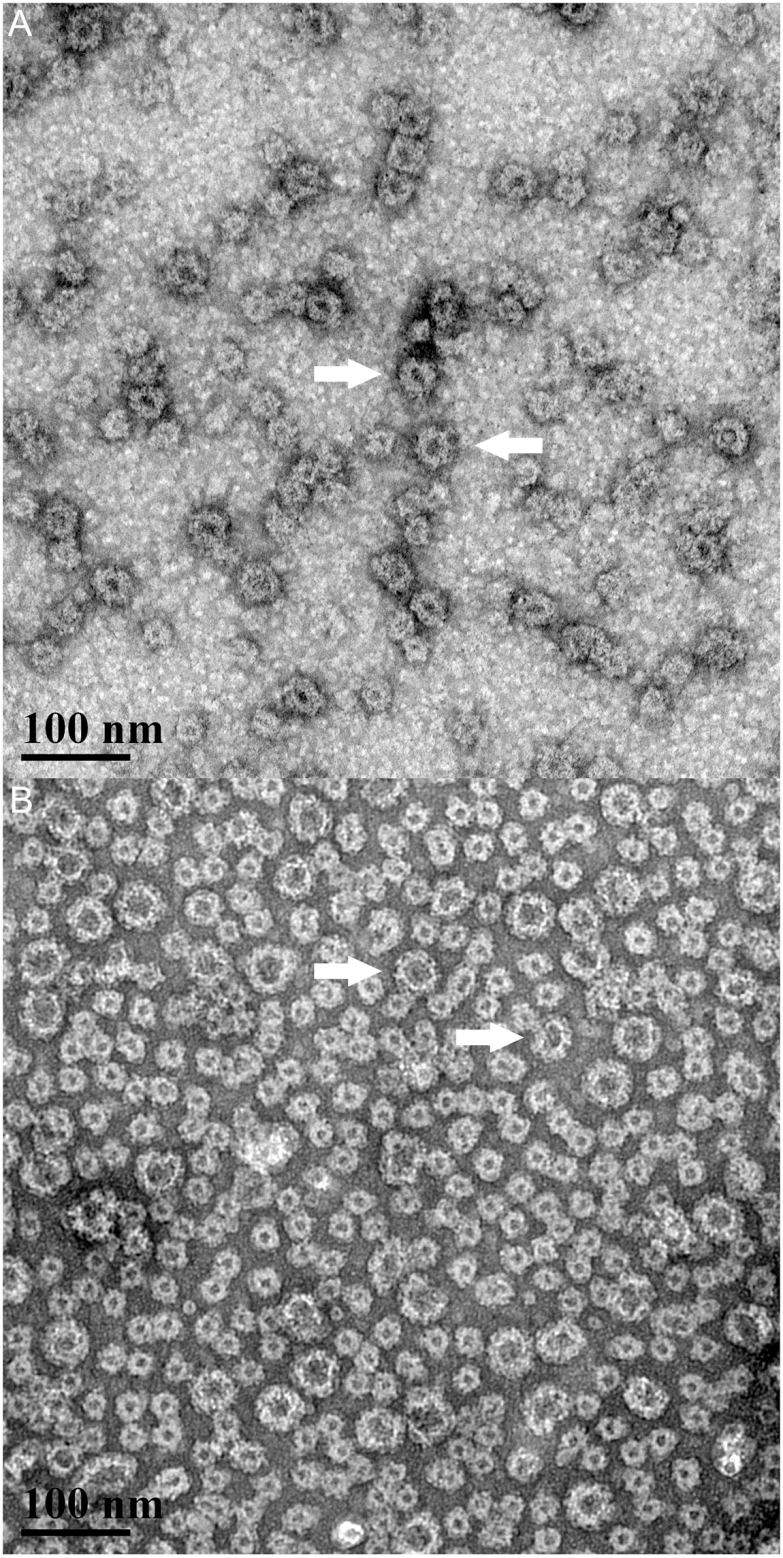
Transmission electron microscopy of norovirus VLPs. GI (A) and GII.4 (B) VLPs were dissolved in water and imaged at 150,000x magnification (scale bar 100 μm). VLP particles were spherical in appearance at the expected size of 23–38 nm.

The GelVac^™^ bivalent nonovirus vaccine powder was manufactured and characterized as previously described and shown to be greater than 95% pure [14]. The analytical characterization of the bivalent vaccine demonstrated that the bivalent powder was as described in previous work [14]. Norovirus GI and GII.4 VLP stocks were analyzed for the presence of intact VLPs by transmission electron microscopy prior to powder formulation. The results confirmed the presence of intact VLPs of the expected sized for both GI (32.6 ± 6 nm) and GII.4 (28.9 ± 9 nm) VLP stocks (Fig 1).

ELISAs were used to confirm the GI or GII.4 VLP dose content in the bivalent vaccine powder. The results showed that the individual powder formulations contained the expected dosage of VLPs and the presence of VLPs in vaccine powders were further confirmed by Western blot (S3B and S3C Fig) [14]. Laser diffraction particle size distribution confirmed the volumetric mean particle size to be 24–42 μm, with the d10 approximately 5–8 μm (Table 2), similar to what was observed previously [14].

**Table 2:** Representative volumetric particle size distribution of GI and GII.4 monovalent and bivalent vaccine powders.

Vaccine powders	Mean (μm)	d10 (μm)	d50 (μm)	d90 (μm)
GI	34.58	7.14	29.74	67.51
GII.4	32.91	6.75	28.43	63.81
0 μg (Placebo)	25.96	4.94	21.81	50.78
5 μg Bivalent	29.49	5.69	24.94	57.60
15 μg Bivalent	33.28	6.78	28.69	64.88
50 μg Bivalent	29.01	5.81	24.74	56.73
100 μg Bivalent	42.02	8.34	36.55	82.86

### Immunogenicity of bivalent GelVac^™^ norovirus vaccine

Dose-dependent immune responses were investigated with bivalent GelVac^™^ vaccine formulations containing GI and GII.4 norovirus VLPs. Animals were dosed on days 0 and 21 with varying amounts of the bivalent norovirus GI and GII.4 VLPs (Table 1). Serum and vaginal lavage samples were collected from the animals on days 0 (pre-immunization), 21 (pre-immunization), 42, and 56. Intestinal lavage was collected upon termination of the study (day 56). No animals became ill or died at any time prior to the experimental endpoint. There was only one animal from the 50 μg dose group that appeared to be a non-responder. The data from this animal was included in the final data analyses.

#### Serum antibody response

**Fig 2:**
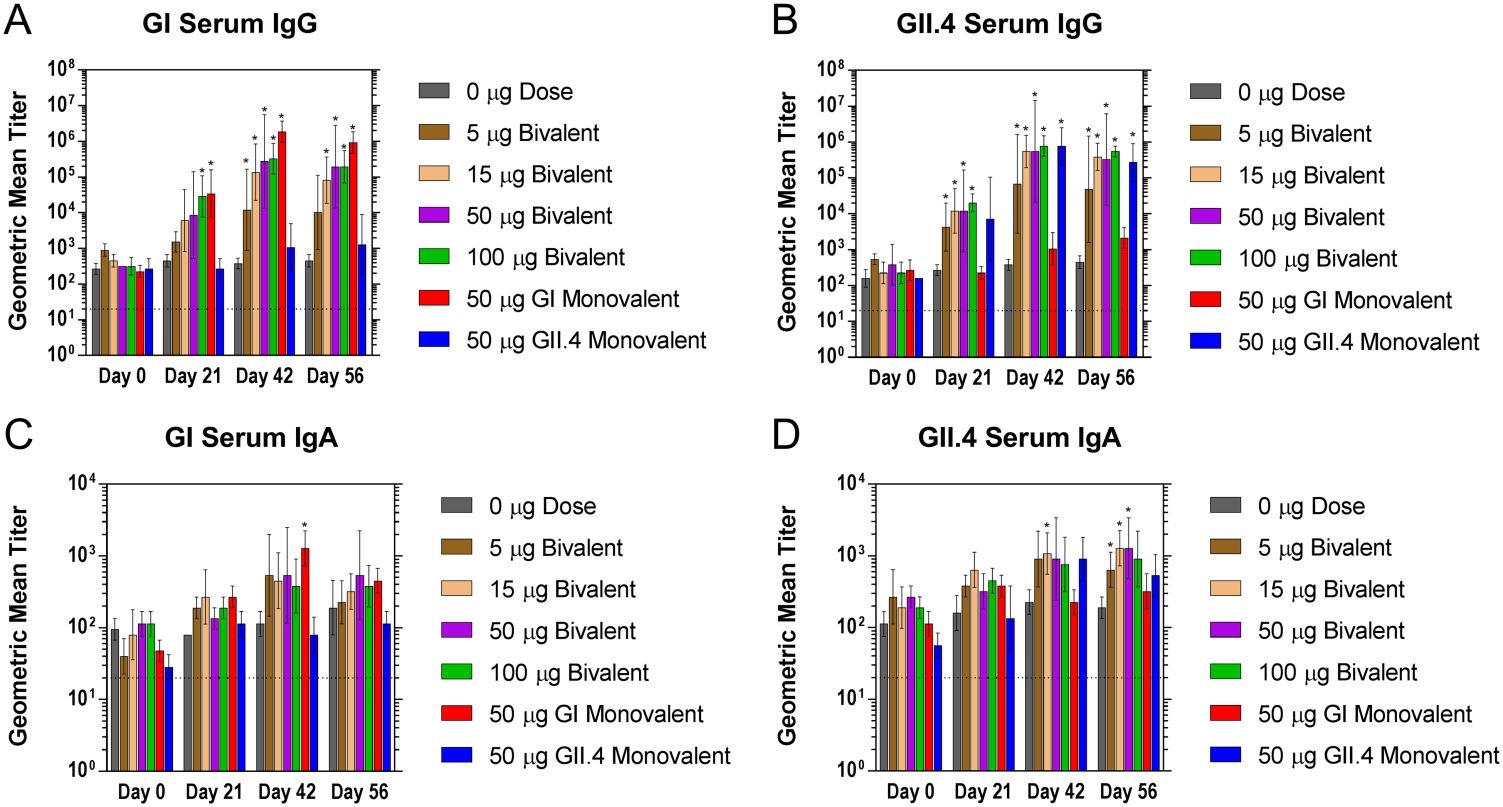
Serum norovirus-specific IgG and IgA production following intranasal immunization with GelVac^™^ bivalent and monovalent vaccine powders. Female Hartley guinea pigs were immunized intranasally with 20 mg of a bivalent vaccine powder formulation containing various amounts of GI and GII.4 VLPs on days 0 and 21. Serum samples were collected on day 0, 14, 21, 42, and 56 and analyzed for specific IgG antibodies against GI (A) and GII.4 (B). Serum samples were also analyzed for specific IgA antibodies against GI (C) and GII.4 (D). Error bars are provided as geometric standard deviation. *p<0.05 as compared to the placebo control group. Horizontal dotted line depicts the limit of detection for these assays.

Serum samples exhibited a dose-dependent increase in total antigen specific IgG antibodies with the bivalent vaccine powder for both GI and GII.4 antigens (Fig 2). IgG titers in serum increased on day 21 and a further increase was observed on day 42 at all doses > 5 μg as compared to the control group. At day 21, a significant difference between the 100 μg dose group and the placebo control was observed (S1–S5 Tables). By day 42 all dose groups containing GI VLP exhibit a significantly higher IgG titer than the placebo and by day 56, all groups ≥ 15 μg exhibit a significant increase over placebo (Fig 2A). No significant differences were observed between the GI monovalent test group and the 50 μg bivalent test group indicating there is likely no appreciable interference from the inclusion of the GII.4 VLPs. By day 42, GI IgG titers increased by >25-fold compared to placebo which was sustained to day 56. Similar results were observed for GII.4 IgG titers with an increase of ≥ 30-fold at day 21 and up to ≥ 700-fold by day 42 (Fig 2B). No significant differences were observed between the GII.4 monovalent test group and the 50 μg bivalent test group indicating there is likely no appreciable interference from the inclusion of the GI VLPs. The lowest dose that elicited an antigen specific IgG response was 5 μg for both GI and GII.4 which corresponded to a titer of 30800 and 245840 on day 56, respectively.

This study also included measurement antigen specific IgA titers in serum by ELISA. At day 42, anti-GI and anti-GII.4 specific VLP IgA antibodies were observed at all doses that were administered when compared to placebo (Fig 2). At day 56, bivalent dose groups of 5, 15, and 50 μg showed a significantly higher titer than the placebo group for the GII.4 antigen. These results demonstrated that the bivalent GI/GII.4 VLP vaccine formulations were highly immunogenic when compared to placebo control.

IgG subclasses IgG1 and IgG2 were measured by ELISA using the pooled serum samples from each group (S4 Fig). GI and GII.4 IgG2 specific titers were shown to be qualitatively higher than GI or GII.4 IgG1 specific titers at day 21. IgG1 and IgG2 boosting effects were observed for both GI and GII.4 at day 42. Overall, the IgG2 titers were higher than IgG1 titers for both GI and GII.4 VLPs. These results show that the bivalent GI/GII.4 VLP vaccine formulations were highly immunogenic relative to the placebo control and capable of producing a wide range of antibody responses.

#### Serum neutralization antibody response

**Fig 3:**
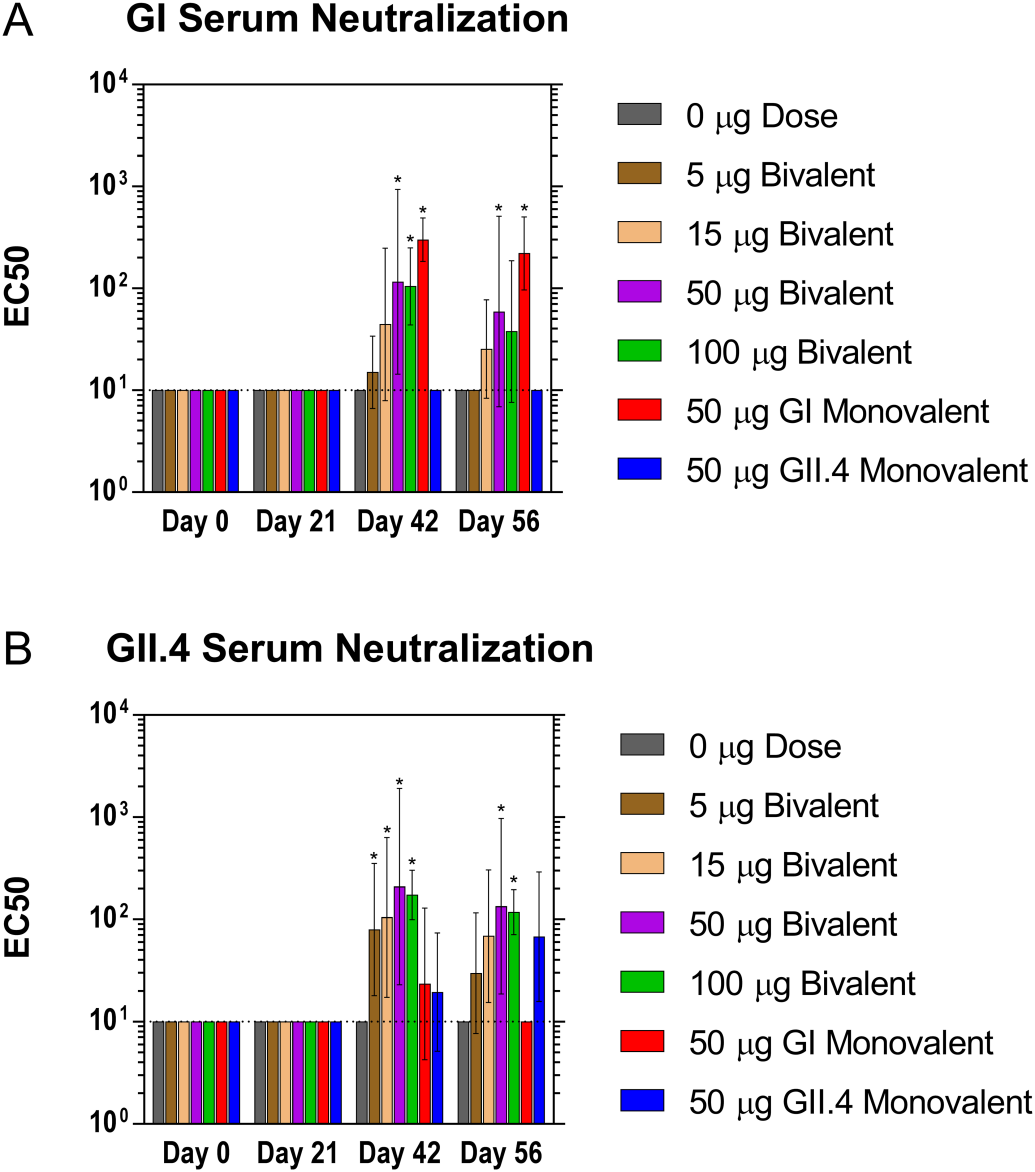
Neutralizing antibody production following intranasal immunization with GelVac^™^ dry powder bivalent and monovalent vaccine. Female Hartley guinea pigs were immunized intranasally with 20 mg of a bivalent vaccine powder formulation containing various amounts of GI and GII.4 VLPs on days 0 and 21. Serum samples were collected on days 0, 14, 21, 42, and 56 and analyzed for GI (A) and GII.4 (B) neutralizing antibodies. Error bars are provided as geometric standard deviation. *p<0.05 as compared to the placebo control group. Horizontal dotted line depicts the limit of detection for these assays.

Antigen specific antibodies were investigated for their ability to inhibit the binding of the norovirus VLPs to porcine gastric mucin. The neutralizing antibodies present in the serum exhibited a dose-dependent response similar to that observed for antigen specific IgG antibody titers (Fig 3). GI neutralizing antibody titers were elevated in the 50 μg and 100 μg dose groups by day 42 with similar titers observed at day 56 as compared to the placebo group. GII.4 neutralizing antibody titers were elevated for all groups by day 42 with similar titers observed at day 56. Comparable to the serum IgG results, the 50 μg bivalent dose group did not show a significant difference from the monovalent dose groups in their respective assays, suggesting that interference in the bivalent formulation is unlikely. By day 42, GI neutralizing antibody titers increased by > 10-fold for all bivalent dose groups ≥ 15 μg and GII.4 neutralizing antibody titers increased by > 14-fold for all dose groups ≥ 5 μg, consistent with the findings with serum IgG titers. The lowest dose that produced a detectable neutralization titer at day 56 was 15 μg for GI and 5 μg for GII.4. The neutralizing antibody titers followed a similar dose-dependent response to that observed for the total serum IgG titers.

#### Mucosal antibody response

**Fig 4:**
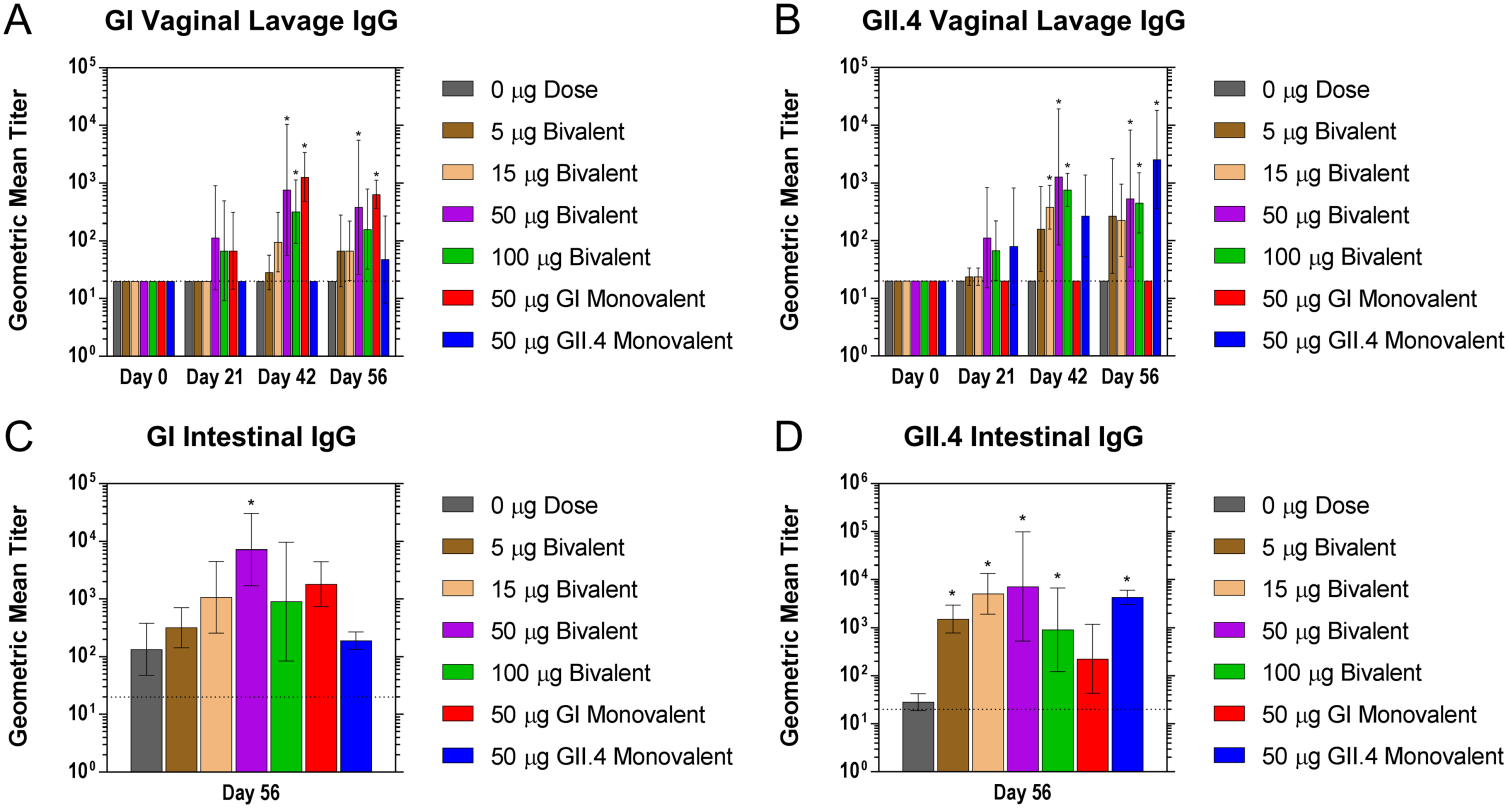
Mucosal norovirus-specific antibody production following intranasal immunization with GelVac^™^ dry powder bivalent and monovalent vaccine. Female Hartley guinea pigs were immunized intranasally with 20 mg of a bivalent vaccine powder formulation containing various amounts of GI and GII.4 VLPs on days 0 and 21. Vaginal lavage samples were collected on days 0, 14, 21, 42, and 56 and analyzed for GI (A) and GII.4 (B) norovirus-specific antibodies. On day 56, animals were euthanized and intestinal lavage samples were analyzed for GI (C) and GII.4 (D) norovirus-specific antibodies. Error bars are provided as geometric standard deviation. *p<0.05 as compared to the placebo control group. Horizontal dotted line depicts the limit of detection for these assays.

Mucosal antibody titers were evaluated in the reproductive tracts and intestines to investigate the mucosal immune response to the bivalent vaccine (Fig 4). GI and GII.4 vaginal IgG antibody titers were elevated in 50 μg and 100 μg dose groups by day 21 when compared to controls, and in all dose groups greater than 5 μg by day 56. GII.4 vaginal antibody titers were elevated in the 5 μg, 15 μg, 50 μg, and 100 μg dose groups by day 42 with significant increases over placebo in bivalent doses ≥ 15μg. Both GI and GII.4 elicited a response in mucosal IgG at the lowest dose group of 5 μg. The highest vaginal antibody titers occurred at 50 μg for both GI and GII.4. These results showed that vaginal IgG antibody titers exhibited a dose-dependent response. GI and GII.4 specific IgG titers were also observed in the intestines at day 56 (Fig 4C and 4D). As shown, antibody titers were observed in all treatment groups for both GI and GII.4 specific antibodies. Significant increases over controls in the 50 μg dose group and ≥ 5 μg dose groups were observed for GI and GII.4, respectively.

## Discussion

Human noroviruses cause acute, debilitating gastroenteritis characterized by vomiting and diarrhea. The US Centers of Disease Control and Prevention estimate it is the leading cause of epidemic non-bacterial gastroenteritis in the US. Multiple vaccine strategies, mostly relying on VLP antigens, have demonstrated proof of efficacy in human challenge studies [18, 44–48]. Previous studies have demonstrated that a norovirus VLP GelVac^™^ dry powder vaccine produces a robust systemic and mucosal immune response [14, 16]. In the present study, we describe the systemic, mucosal, and neutralizing antibody responses to a GelVac^™^ dry powder bivalent norovirus vaccine containing varying amounts of both GI and GII.4 norovirus VLP antigens.

Guinea pigs were used in this study due to the intranasal powder delivery system that we employed. Previous studies [16] have used six animals per group and demonstrated similar levels of significance as demonstrated in the present study. In addition, studies by Baric and his laboratory also used four animals per group [49] and our previous work [14]. For these reasons, four animals per group were used throughout this study.

The results depict that the bivalent vaccine powder induced a dose-dependent antibody response against GI and GII.4 VLPs without exhibiting interference from either antigen. Antigen specific IgG antibody titers present in serum samples increased with greater amounts of both GI and GII.4 VLP antigens present in the powder and reached a maximal level at 50 μg of VLP antigen. Higher doses of VLPs did not result in significantly higher titers of antigen specific IgGs in either serum or mucosal. Additionally, a boosting effect on systemic and mucosal antigen specific IgGs and IgAs was observed for each VLP antigen after the second powder administration on day 21.

A surrogate marker for efficacy identified that correlates well with protection in humans and chimpanzees is the production of antibodies that neutralize HBGA binding sites [31, 35, 50]. In particular, previous studies have shown the levels of IgA in mucosal tissues was strongly correlated with the blocking activity, suggesting that IgA, but not IgG, was the major norovirus blocking antibody on the mucosal surface [36]. In addition, it has been shown that only mucosal immunization induces the development of functional anti-norovirus IgA on mucosal surfaces [36]. The data presented herein demonstrated that the GelVac^™^ bivalent norovirus intranasal vaccine powder containing both GI and GII.4 VLPs was capable of producing antibodies that inhibited the binding of the VLPs to pig gastric mucin. A dose-dependence was observed for these neutralizing antibodies with the amount of GI or GII.4 VLP administered. Furthermore, neutralizing antibody titers demonstrated a boosting effect after the second dose in a similar fashion as observed for serum IgG antibodies. An increased VLP antigen dose was required for the production of neutralizing antibody titers as compared to the induction of total specific IgG antibodies. A dose of at least 50 μg for GII.4 and 50 μg for GI VLP antigen was required to raise the production of neutralizing antibodies.

Antigen specific mucosal antibody titers were determined using vaginal lavage sampling. Antigen specific IgG antibody production in the vaginal lavages showed similar trends as those observed in the intestinal tract. The antigen specific antibodies observed in these mucosal tissues also showed similar trends to titers observed in serum for both antigen specific IgG antibodies and neutralizing antibodies. IgG antibodies in the mucosa are most likely conferred through transudation of serum IgG antibodies [51]. A general link between increased serum IgG with increased vaginal lavage IgG is observed, as well as an increase in serum IgA [16, 52]. This link, coupled with the observed overall decrease in signal in vaginal lavage samples and IgA detection could contribute to mucosal IgA titers falling below the assay limit of detection. Additionally, the reagents used in previous work were not available for this study and make comparisons challenging. These results suggest that our GelVac^™^ vaccine powder is capable of inducing a mucosal response along with a neutralizing antibody response.

There are currently no licensed vaccines for norovirus. There are many factors that complicate the development of norovirus vaccines including the lack of appropriate model systems to explore vaccine target efficacy, unknown duration of protective immunity, antigenic variation and drift within genogroups and genotypes, and the unknown effects of pre-exposure history. The recent demonstration of the cultivation of norovirus in cell culture should permit human host pathogen interactions and allow assessment of various methods to prevent and treat norovirus infections [29]. Despite these limitations, vaccine feasibility has been demonstrated using norovirus VLPs in human challenge studies [17, 18]. However, these studies demonstrated only 47% efficacy in a 2-dose intranasal VLP vaccine and 52% protection in an IM administration against all severity levels of disease (*p* = 0.028) and 68% (*p* = 0.068) against moderate to severe disease. These vaccines were formulated with Aluminum Hydroxide and Monophosphoryl lipid adjuvants. These vaccines are still in clinical development. A norovirus-rotavirus protein combination vaccine is in preclinical development with promising results [15]. In addition, a novel combination construct which contains VLPs of norovirus GII.4 and enterovirus 71 has been shown to elicit functional antibodies to both viruses without evidence of interference [53]. One other intranasal norovirus vaccine is currently in development. This is based on the norovirus P particle derived from the protruding (P) domain of the norovirus VP1 capsid protein being delivered intranasally in gnotobiotic pigs [54]. The study demonstrated cross-variant protection of the P particle vaccine against human noroviral diarrhea. Despite these advances, other formulations of norovirus vaccine, such as the one described in this study, may provide advantages over the vaccines currently in development. One advantage of the current formulation over other vaccine candidates is the lack of adjuvants. The current GelVac^™^ is made from materials that the FDA has considered generally regarded as safe (GRAS). The data presented herein, as well as previous work [14, 16], demonstrate that the current vaccine candidate does not require the addition of adjuvants and is administered IN. Current studies being performed with the GelVac^™^ dry powder norovirus vaccine include a comparison of IN and IM administration.

The data presented in this study using the GelVac^™^ dry powder bivalent norovirus vaccine extends the results from previous studies [14, 16]. The data demonstrates that there was no inhibition or competition between the two VLP antigens in the bivalent GelVac^™^ nasal powder on the production of systemic and mucosal anti-norovirus specific antibodies. A dose content of 15–50 μg GI or GII.4 VLP antigen appeared to be required to produce a robust response. Future studies will be conducted with GelVac^™^ GI and GII.4 bivalent vaccine formulation administered either intranasally or intramuscularly. The results will help to demonstrate the efficacy of the intranasal formulation when compared to an intramuscular injection and determine the antigen dose for this bivalent vaccine that could be used in additional pre-clinical development.

## Supporting information

S1 Fig Thermo stability evaluation of norovirus VLPs using SYPRO orange. VLPs were diluted in 4x SYPRO orange solution and the melt curve was analyzed using a fluorescent thermocycler. Data is plotted as the change in Fluorescence per unit Temperature. A. Norovirus GI VLP melt curve. B. Norovirus GII.4 VLP melt curve.
(TIF)

S2 Fig Assessment of capture ELISA specificity to intact VLPs. VLP samples (0.2 μg/mL) were treated at varying temperatures for 5 minutes. Each sample was then analyzed by capture ELISA.
(TIF)

S3 Fig SDS-PAGE and western blot analysis of GI and GII.4 VLP in norovirus vaccine powders. (A) (SDS-PAGE), (B) GI VLP (western blot), and (C) GII.4 VLP (western blot).
(TIF)

S4 Fig Serum norovirus-specific IgG1 and IgG2 production following intranasal administration with GelVac^™^ dry powder bivalent and monovalent vaccine. Serum samples were analyzed for norovirus-specific IgG1 antibodies against GI (A) and GII.4 (B), and norovirus-specific IgG2 antibodies against GI (C) and GII.4 (D). Horizontal dotted line depicts the limit of detection for these assays.
(TIF)

S1 Table List of multiple comparison adjusted p-values for Tukey’s post hoc test for significance following a two-way analysis of variance. (DOCX)

S2 Table Total antigen specific serum IgG, IgA and neutralizing antibody titers. (DOCX)

S3 Table Total antigen specific serum IgG1 and IgG2 antibody titers. (DOCX)

S4 Table Total antigen specific IgG titers from intestinal lavage samples. (DOCX)

S5 Table Total antigen specific IgG titers from vaginal lavage samples. (DOCX)
