# Supplementary figures and images for: Intranasal delivery of a bivalent norovirus vaccine formulated in an in situ gelling dry powder
Source: PLoS One. 2017 May 18;12(5):e0177310. doi: 10.1371/journal.pone.0177310 (PMC5436670; doi:10.1371/journal.pone.0177310)

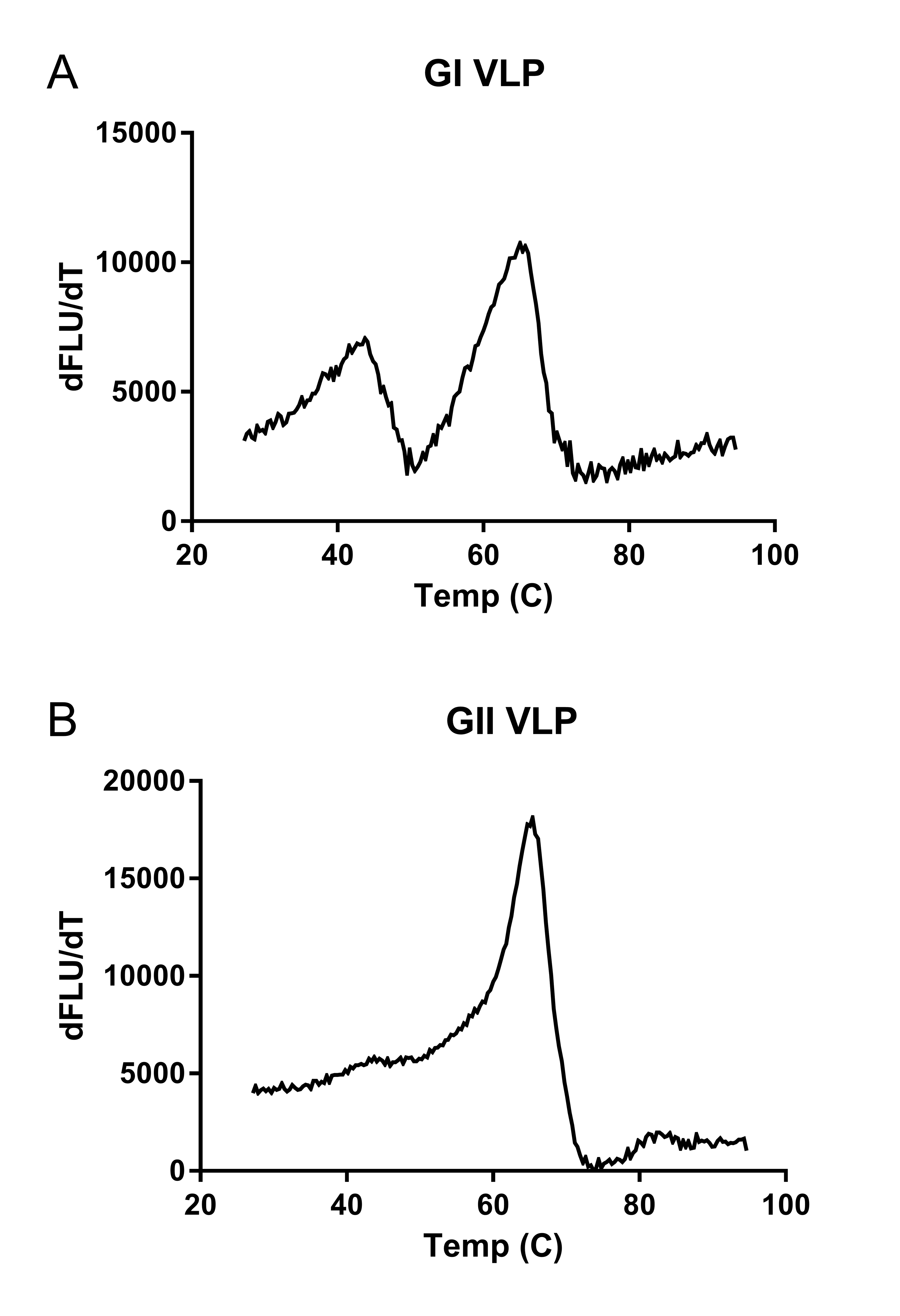

Supplement: S1 Fig — VLPs were diluted in 4x SYPRO orange solution and the melt curve was analyzed using a fluorescent thermocycler. Data is plotted as the change in Fluorescence per unit Temperature. A. Norovirus GI VLP melt curve. B. Norovirus GII.4 VLP melt curve. (TIF) [file pone.0177310.s001.tif]

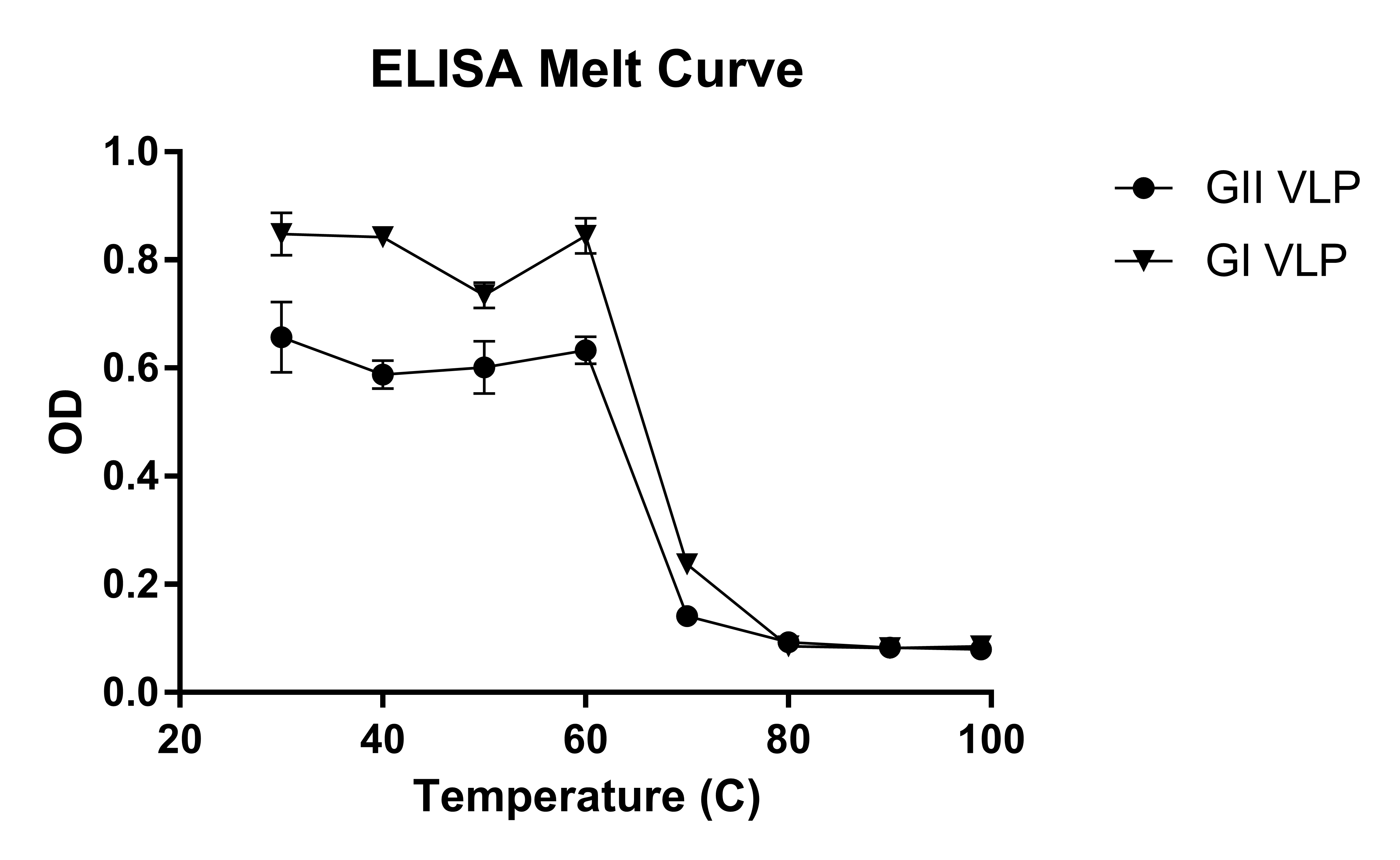

Supplement: S2 Fig — VLP samples (0.2 μg/mL) were treated at varying temperatures for 5 minutes. Each sample was then analyzed by capture ELISA. (TIF) [file pone.0177310.s002.tif]

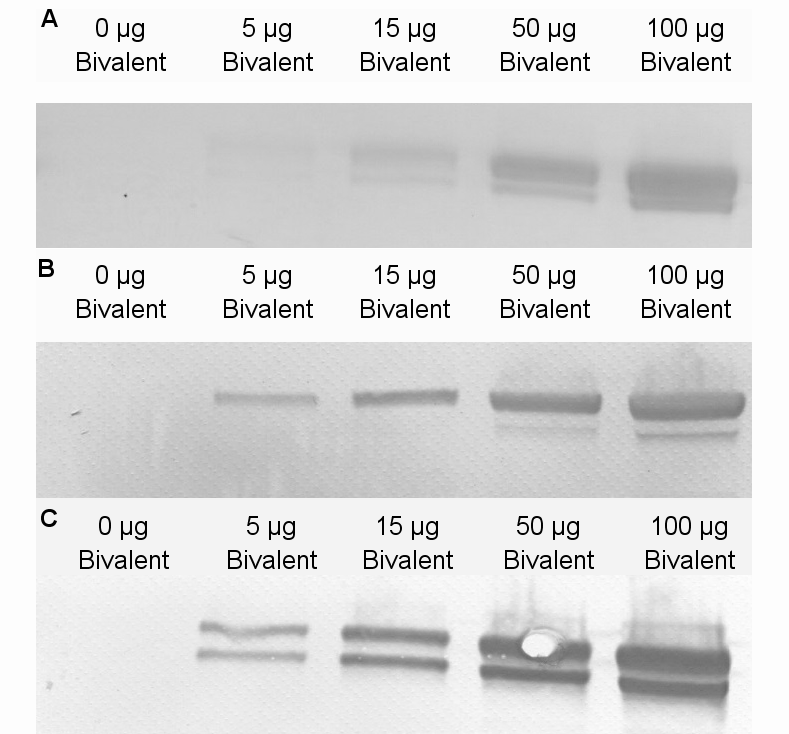

Supplement: S3 Fig — (A) (SDS-PAGE), (B) GI VLP (western blot), and (C) GII.4 VLP (western blot). (TIF) [file pone.0177310.s003.tif]

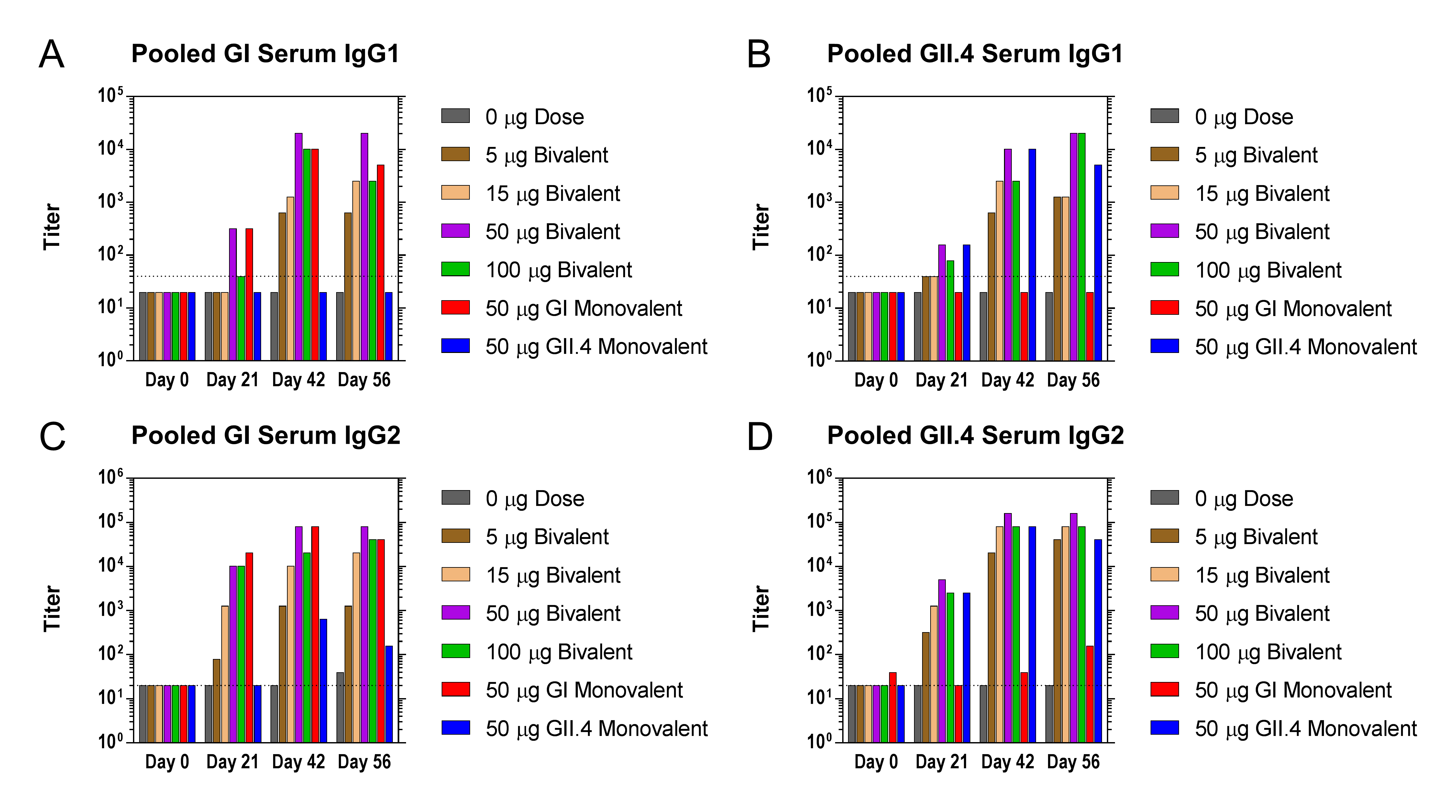

Supplement: S4 Fig — Serum samples were analyzed for norovirus-specific IgG1 antibodies against GI (A) and GII.4 (B), and norovirus-specific IgG2 antibodies against GI (C) and GII.4 (D). Horizontal dotted line depicts the limit of detection for these assays. (TIF) [file pone.0177310.s004.tif]
