# Supplementary material for: Intranasal delivery of a bivalent norovirus vaccine formulated in an in situ gelling dry powder
Source: PLoS One. 2017 May 18;12(5):e0177310. doi: 10.1371/journal.pone.0177310 (PMC5436670; doi:10.1371/journal.pone.0177310)
Supplement: S1 Table — (DOCX) [file pone.0177310.s005.docx]

| Assay | Day | Comparison | Adjusted p-Value |
| --- | --- | --- | --- |
| GI IgG Serum | 21 | 0 µg Dose vs. 50 µg GI Monovalent | 0.001716924 |
| GI IgG Serum | 21 | 0 µg Dose vs. 100 µg Bivalent | 0.003020039 |
| GI IgG Serum | 21 | 50 µg GI Monovalent vs. 50 µg Bivalent | 0.843002262 |
| GI IgG Serum | 42 | 0 µg Dose vs. 50 µg GI Monovalent | 3E-10 |
| GI IgG Serum | 42 | 0 µg Dose vs. 5 µg Bivalent | 0.023884064 |
| GI IgG Serum | 42 | 0 µg Dose vs. 15 µg Bivalent | 5.6499E-06 |
| GI IgG Serum | 42 | 0 µg Dose vs. 50 µg Bivalent | 3.417E-07 |
| GI IgG Serum | 42 | 0 µg Dose vs. 100 µg Bivalent | 1.666E-07 |
| GI IgG Serum | 42 | 50 µg GI Monovalent vs. 50 µg Bivalent | 0.546270779 |
| GI IgG Serum | 56 | 0 µg Dose vs. 50 µg GI Monovalent | 4.4E-09 |
| GI IgG Serum | 56 | 0 µg Dose vs. 15 µg Bivalent | 8.10271E-05 |
| GI IgG Serum | 56 | 0 µg Dose vs. 50 µg Bivalent | 2.8345E-06 |
| GI IgG Serum | 56 | 0 µg Dose vs. 100 µg Bivalent | 2.8345E-06 |
| GI IgG Serum | 56 | 50 µg GI Monovalent vs. 50 µg Bivalent | 0.756000982 |
| GII IgG Serum | 21 | 0 µg Dose vs. 15 µg Bivalent | 0.01752063 |
| GII IgG Serum | 21 | 0 µg Dose vs. 50 µg Bivalent | 0.01752063 |
| GII IgG Serum | 21 | 0 µg Dose vs. 100 µg Bivalent | 0.004071094 |
| GII IgG Serum | 21 | 50 µg GII.4 Monovalent vs. 50 µg Bivalent | 0.999223206 |
| GII IgG Serum | 42 | 0 µg Dose vs. 50 µg GII.4 Monovalent | 3.16E-08 |
| GII IgG Serum | 42 | 0 µg Dose vs. 5 µg Bivalent | 0.000261218 |
| GII IgG Serum | 42 | 0 µg Dose vs. 15 µg Bivalent | 1.239E-07 |
| GII IgG Serum | 42 | 0 µg Dose vs. 50 µg Bivalent | 1.239E-07 |
| GII IgG Serum | 42 | 0 µg Dose vs. 100 µg Bivalent | 3.16E-08 |
| GII IgG Serum | 42 | 50 µg GII.4 Monovalent vs. 50 µg Bivalent | 0.999926063 |
| GII IgG Serum | 56 | 0 µg Dose vs. 50 µg GII.4 Monovalent | 3.4461E-06 |
| GII IgG Serum | 56 | 0 µg Dose vs. 5 µg Bivalent | 0.001415799 |
| GII IgG Serum | 56 | 0 µg Dose vs. 15 µg Bivalent | 0.000000928 |
| GII IgG Serum | 56 | 0 µg Dose vs. 50 µg Bivalent | 1.7944E-06 |
| GII IgG Serum | 56 | 0 µg Dose vs. 100 µg Bivalent | 2.437E-07 |
| GII IgG Serum | 56 | 50 µg GII.4 Monovalent vs. 50 µg Bivalent | 0.999998787 |
| GI IgA Serum | 21 | 50 µg GI Monovalent vs. 50 µg Bivalent | 0.8047810477 |
| GI IgA Serum | 42 | 0 µg Dose vs. 50 µg GI Monovalent | 0.0001029417 |
| GI IgA Serum | 42 | 50 µg GI Monovalent vs. 50 µg Bivalent | 0.5918917704 |
| GI IgA Serum | 56 | 50 µg GI Monovalent vs. 50 µg Bivalent | 0.9998506279 |
| GII IgA Serum | 21 | 50 µg GII.4 Monovalent vs. 50 µg Bivalent | 0.5152279887 |
| GII IgA Serum | 42 | 0 µg Dose vs. 15 µg Bivalent | 0.0205447748 |
| GII IgA Serum | 42 | 50 µg GII.4 Monovalent vs. 50 µg Bivalent | >0.9999999999 |
| GII IgA Serum | 56 | 0 µg Dose vs. 15 µg Bivalent | 0.0018927356 |
| GII IgA Serum | 56 | 0 µg Dose vs. 50 µg Bivalent | 0.0018927356 |
| GII IgA Serum | 56 | 0 µg Dose vs. 100 µg Bivalent | 0.0205447748 |
| GII IgA Serum | 56 | 50 µg GII.4 Monovalent vs. 50 µg Bivalent | 0.5152279887 |
| GI Neutralization Serum | 21 | 50 µg GI Monovalent vs. 50 µg Bivalent | >0.9999999999 |
| GI Neutralization Serum | 42 | 0 µg Dose vs. 50 µg GI Monovalent | 1.0696E-06 |
| GI Neutralization Serum | 42 | 0 µg Dose vs. 50 µg Bivalent | 0.000837352 |
| GI Neutralization Serum | 42 | 0 µg Dose vs. 100 µg Bivalent | 0.001597267 |
| GI Neutralization Serum | 42 | 50 µg GI Monovalent vs. 50 µg Bivalent | 0.637180679 |
| GI Neutralization Serum | 56 | 0 µg Dose vs. 50 µg GI Monovalent | 1.04884E-05 |
| GI Neutralization Serum | 56 | 0 µg Dose vs. 50 µg Bivalent | 0.037293616 |
| GI Neutralization Serum | 56 | 50 µg GI Monovalent vs. 50 µg Bivalent | 0.251340536 |
| GI Neutralization Serum | 56 | 5 µg Bivalent vs. 100 µg Bivalent | 0.239878167 |
| GII Neutralization Serum | 21 | 50 µg GII.4 Monovalent vs. 50 µg Bivalent | >0.9999999999 |
| GII Neutralization Serum | 42 | 0 µg Dose vs. 5 µg Bivalent | 0.044520189 |
| GII Neutralization Serum | 42 | 0 µg Dose vs. 15 µg Bivalent | 0.013962626 |
| GII Neutralization Serum | 42 | 0 µg Dose vs. 50 µg Bivalent | 0.000435475 |
| GII Neutralization Serum | 42 | 0 µg Dose vs. 100 µg Bivalent | 0.001189696 |
| GII Neutralization Serum | 42 | 50 µg GII.4 Monovalent vs. 50 µg Bivalent | 0.012456648 |
| GII Neutralization Serum | 56 | 0 µg Dose vs. 50 µg Bivalent | 0.004320732 |
| GII Neutralization Serum | 56 | 0 µg Dose vs. 100 µg Bivalent | 0.008303382 |
| GII Neutralization Serum | 56 | 50 µg GII.4 Monovalent vs. 50 µg Bivalent | 0.949388761 |
| GII Neutralization Serum | 56 | 5 µg Bivalent vs. 100 µg Bivalent | 0.4095819 |
| GI IgG Vaginal | 21 | 50 µg GI Monovalent vs. 50 µg Bivalent | 0.995720055 |
| GI IgG Vaginal | 42 | 0 µg Dose vs. 50 µg GI Monovalent | 5.73026E-05 |
| GI IgG Vaginal | 42 | 0 µg Dose vs. 50 µg Bivalent | 0.000626093 |
| GI IgG Vaginal | 42 | 0 µg Dose vs. 100 µg Bivalent | 0.020135318 |
| GI IgG Vaginal | 42 | 50 µg GI Monovalent vs. 50 µg Bivalent | 0.995720055 |
| GI IgG Vaginal | 56 | 0 µg Dose vs. 50 µg GI Monovalent | 0.00132737 |
| GI IgG Vaginal | 56 | 0 µg Dose vs. 50 µg Bivalent | 0.010704404 |
| GI IgG Vaginal | 56 | 50 µg GI Monovalent vs. 50 µg Bivalent | 0.995720055 |
| GII IgG Vaginal | 21 | 50 µg GII.4 Monovalent vs. 50 µg Bivalent | 0.999729724 |
| GII IgG Vaginal | 42 | 0 µg Dose vs. 15 µg Bivalent | 0.024826572 |
| GII IgG Vaginal | 42 | 0 µg Dose vs. 50 µg Bivalent | 0.000266102 |
| GII IgG Vaginal | 42 | 0 µg Dose vs. 100 µg Bivalent | 0.00215054 |
| GII IgG Vaginal | 56 | 0 µg Dose vs. 50 µg GII.4 Monovalent | 1.27198E-05 |
| GII IgG Vaginal | 56 | 0 µg Dose vs. 50 µg Bivalent | 0.00772019 |
| GII IgG Vaginal | 56 | 0 µg Dose vs. 100 µg Bivalent | 0.014051235 |
| GII IgG Vaginal | 56 | 50 µg GII.4 Monovalent vs. 50 µg Bivalent | 0.595821289 |
| GI IgG IL | 56 | 0 µg Dose vs. 50 µg Bivalent | 0.005715887 |
| GI IgG IL | 56 | 50 µg GI Monovalent vs. 50 µg Bivalent | 0.755908514 |
| GII IgG IL | 56 | 0 µg Dose vs. 50 µg GII.4 Monovalent | 0.001561468 |
| GII IgG IL | 56 | 0 µg Dose vs. 5 µg Bivalent | 0.014899519 |
| GII IgG IL | 56 | 0 µg Dose vs. 15 µg Bivalent | 0.001068315 |
| GII IgG IL | 56 | 0 µg Dose vs. 50 µg Bivalent | 0.000501191 |
| GII IgG IL | 56 | 0 µg Dose vs. 100 µg Bivalent | 0.043660364 |
| GII IgG IL | 56 | 50 µg GII.4 Monovalent vs. 50 µg Bivalent | 0.998621876 |
