# Supplementary material for: Intranasal delivery of a bivalent norovirus vaccine formulated in an in situ gelling dry powder
Source: PLoS One. 2017 May 18;12(5):e0177310. doi: 10.1371/journal.pone.0177310 (PMC5436670; doi:10.1371/journal.pone.0177310)
Supplement: S2 Table — (DOCX) [file pone.0177310.s006.docx]

| Animal ID | Antigen | Antigen Dose | Sample Point | GI VLP | | | GII.4 VLP | | |
| --- | --- | --- | --- | --- | --- | --- | --- | --- | --- |
|  |  |  |  | IgG Titer | IgA Titer | Neutralizing Titer | IgG Titer | IgA Titer | Neutralizing Titer |
|  |  |  |  |  |  |  |  |  |  |
|  |  |  |  |  |  |  |  |  |  |
| GP52 | Bivalent Vaccine | 0 µg | Day 0 - Serum | 160 | 160 | 10 | 160 | 160 | 10 |
|  |  |  | Day 21 - Serum | 320 | 80 | 10 | 320 | 320 | 10 |
|  |  |  | Day 42 - Serum | 320 | 80 | 10 | 320 | 320 | 10 |
|  |  |  | Day 56 - Serum | 320 | 160 | 10 | 640 | 320 | 10 |
| GP53 | Bivalent Vaccine | 0 µg | Day 0 - Serum | 320 | 80 | 10 | 80 | 80 | 10 |
|  |  |  | Day 21 - Serum | 640 | 80 | 10 | 160 | 80 | 10 |
|  |  |  | Day 42 - Serum | 640 | 160 | 10 | 640 | 160 | 10 |
|  |  |  | Day 56 - Serum | 640 | 640 | 10 | 320 | 160 | 10 |
| GP54 | Bivalent Vaccine | 0 µg | Day 0 - Serum | 320 | 80 | 10 | 160 | 80 | 10 |
|  |  |  | Day 21 - Serum | 640 | 80 | 10 | 320 | 160 | 10 |
|  |  |  | Day 42 - Serum | 320 | 160 | 10 | 320 | 160 | 10 |
|  |  |  | Day 56 - Serum | 640 | 160 | 10 | 320 | 160 | 10 |
| GP55 | Bivalent Vaccine | 0 µg | Day 0 - Serum | 320 | 80 | 10 | 320 | 160 | 10 |
|  |  |  | Day 21 - Serum | 320 | 80 | 10 | 320 | 160 | 10 |
|  |  |  | Day 42 - Serum | 320 | 80 | 10 | 320 | 320 | 10 |
|  |  |  | Day 56 - Serum | 320 | 80 | 10 | 640 | 160 | 10 |
| GP56 | GI Vaccine | 50 µg | Day 0 - Serum | 320 | 80 | 10 | 160 | 160 | 10 |
|  |  |  | Day 21 - Serum | 81920 | 320 | 10 | 160 | 320 | 10 |
|  |  |  | Day 42 - Serum | 5242880 | 2560 | 577.142 | 5120 | 320 | 303.325 |
|  |  |  | Day 56 - Serum | 2621440 | 640 | 626.715 | 5120 | 320 | 10 |
| GP57 | GI Vaccine | 50 µg | Day 0 - Serum | 160 | 40 | 10 | 160 | 80 | 10 |
|  |  |  | Day 21 - Serum | 5120 | 160 | 10 | 160 | 320 | 10 |
|  |  |  | Day 42 - Serum | 1310720 | 640 | 249.241 | 640 | 160 | 10 |
|  |  |  | Day 56 - Serum | 655360 | 320 | 198.666 | 2560 | 320 | 10 |
| GP58 | GI Vaccine | 50 µg | Day 0 - Serum | 160 | 40 | 10 | 320 | 80 | 10 |
|  |  |  | Day 21 - Serum | 163840 | 320 | 10 | 320 | 320 | 10 |
|  |  |  | Day 42 - Serum | 1310720 | 1280 | 314.78 | 640 | 160 | 10 |
|  |  |  | Day 56 - Serum | 655360 | 640 | 226.049 | 1280 | 160 | 10 |
| GP59 | GI Vaccine | 50 µg | Day 0 - Serum | 320 | 40 | 10 | 640 | 160 | 10 |
|  |  |  | Day 21 - Serum | 20480 | 320 | 10 | 320 | 640 | 10 |
|  |  |  | Day 42 - Serum | 1310720 | 1280 | 179.451 | 640 | 320 | 10 |
|  |  |  | Day 56 - Serum | 655360 | 320 | 84.016 | 1280 | 320 | 10 |
| GP60 | GII Vaccine | 50 µg | Day 0 - Serum | 320 | 40 | 10 | 160 | 40 | 10 |
|  |  |  | Day 21 - Serum | 320 | 160 | 10 | 163840 | 640 | 10 |
|  |  |  | Day 42 - Serum | 10240 | 80 | 10 | 2621440 | 1280 | 10 |
|  |  |  | Day 56 - Serum | 20480 | 80 | 10 | 1310720 | 1280 | 329.326 |
| GP61 | GII Vaccine | 50 µg | Day 0 - Serum | 160 | 40 | 10 | 160 | 80 | 10 |
|  |  |  | Day 21 - Serum | 640 | 160 | 10 | 20480 | 80 | 10 |
|  |  |  | Day 42 - Serum | 640 | 160 | 10 | 1310720 | 1280 | 10 |
|  |  |  | Day 56 - Serum | 320 | 160 | 10 | 327680 | 640 | 106.737 |
| GP62 | GII Vaccine | 50 µg | Day 0 - Serum | 160 | 20 | 10 | 160 | 40 | 10 |
|  |  |  | Day 21 - Serum | 160 | 80 | 10 | 2560 | 80 | 10 |
|  |  |  | Day 42 - Serum | 320 | 40 | 10 | 655360 | 320 | 144.178 |
|  |  |  | Day 56 - Serum | 1280 | 160 | 10 | 163840 | 320 | 60.077 |
| GP63 | GII Vaccine | 50 µg | Day 0 - Serum | 640 | 20 | 10 | 160 | 80 | 10 |
|  |  |  | Day 21 - Serum | 160 | 80 | 10 | 320 | 80 | 10 |
|  |  |  | Day 42 - Serum | 640 | 80 | 10 | 163840 | 1280 | 10 |
|  |  |  | Day 56 - Serum | 320 | 80 | 10 | 81920 | 320 | 10 |
| GP64 | Bivalent Vaccine | 5 µg | Day 0 - Serum | 1280 | 40 | 10 | 640 | 320 | 10 |
|  |  |  | Day 21 - Serum | 1280 | 320 | 10 | 2560 | 320 | 10 |
|  |  |  | Day 42 - Serum | 20480 | 1280 | 10 | 327680 | 1280 | 87.531 |
|  |  |  | Day 56 - Serum | 20480 | 640 | 10 | 163840 | 640 | 10 |
| GP65 | Bivalent Vaccine | 5 µg | Day 0 - Serum | 1280 | 80 | 10 | 640 | 640 | 10 |
|  |  |  | Day 21 - Serum | 640 | 160 | 10 | 640 | 320 | 10 |
|  |  |  | Day 42 - Serum | 320 | 80 | 10 | 640 | 320 | 10 |
|  |  |  | Day 56 - Serum | 320 | 160 | 10 | 320 | 320 | 10 |
| GP66 | Bivalent Vaccine | 5 µg | Day 0 - Serum | 640 | 40 | 10 | 640 | 320 | 10 |
|  |  |  | Day 21 - Serum | 2560 | 160 | 10 | 20480 | 320 | 10 |
|  |  |  | Day 42 - Serum | 20480 | 1280 | 10 | 163840 | 640 | 142.983 |
|  |  |  | Day 56 - Serum | 20480 | 160 | 10 | 163840 | 640 | 48.713 |
| GP67 | Bivalent Vaccine | 5 µg | Day 0 - Serum | 640 | 20 | 10 | 320 | 80 | 10 |
|  |  |  | Day 21 - Serum | 2560 | 160 | 10 | 10240 | 640 | 10 |
|  |  |  | Day 42 - Serum | 163840 | 640 | 51.056 | 655360 | 2560 | 323.551 |
|  |  |  | Day 56 - Serum | 81920 | 160 | 36.669 | 655360 | 640 | 163.91 |
| GP68 | Bivalent Vaccine | 15 µg | Day 0 - Serum | 320 | 40 | 10 | 160 | 80 | 10 |
|  |  |  | Day 21 - Serum | 320 | 80 | 10 | 2560 | 320 | 10 |
|  |  |  | Day 42 - Serum | 10240 | 320 | 10 | 327680 | 640 | 166.508 |
|  |  |  | Day 56 - Serum | 10240 | 160 | 10 | 327680 | 1280 | 65.871 |
| GP69 | Bivalent Vaccine | 15 µg | Day 0 - Serum | 320 | 40 | 10 | 160 | 160 | 10 |
|  |  |  | Day 21 - Serum | 20480 | 320 | 10 | 40960 | 640 | 10 |
|  |  |  | Day 42 - Serum | 655360 | 1280 | 222.529 | 2621440 | 2560 | 768.502 |
|  |  |  | Day 56 - Serum | 327680 | 320 | 89.825 | 1310720 | 2560 | 376.224 |
| GP70 | Bivalent Vaccine | 15 µg | Day 0 - Serum | 640 | 160 | 10 | 160 | 320 | 10 |
|  |  |  | Day 21 - Serum | 20480 | 640 | 10 | 40960 | 640 | 10 |
|  |  |  | Day 42 - Serum | 163840 | 160 | 10 | 327680 | 640 | 10 |
|  |  |  | Day 56 - Serum | 81920 | 320 | 10 | 327680 | 1280 | 90.485 |
| GP71 | Bivalent Vaccine | 15 µg | Day 0 - Serum | 640 | 160 | 10 | 640 | 320 | 10 |
|  |  |  | Day 21 - Serum | 10240 | 320 | 10 | 5120 | 1280 | 10 |
|  |  |  | Day 42 - Serum | 327680 | 640 | 174.089 | 327680 | 1280 | 94.781 |
|  |  |  | Day 56 - Serum | 163840 | 640 | 46.962 | 163840 | 640 | 10 |
| GP72 | Bivalent Vaccine | 50 µg | Day 0 - Serum | 320 | 80 | 10 | 2560 | 160 | 10 |
|  |  |  | Day 21 - Serum | 2560 | 80 | 10 | 10240 | 320 | 10 |
|  |  |  | Day 42 - Serum | 163840 | 320 | 50.565 | 655360 | 640 | 179.481 |
|  |  |  | Day 56 - Serum | 163840 | 160 | 10 | 327680 | 320 | 84.511 |
| GP73 | Bivalent Vaccine | 50 µg | Day 0 - Serum | 320 | 80 | 10 | 160 | 320 | 10 |
|  |  |  | Day 21 - Serum | 320 | 160 | 10 | 320 | 160 | 10 |
|  |  |  | Day 42 - Serum | 5120 | 80 | 10 | 5120 | 160 | 10 |
|  |  |  | Day 56 - Serum | 5120 | 160 | 10 | 5120 | 320 | 10 |
| GP74 | Bivalent Vaccine | 50 µg | Day 0 - Serum | 320 | 160 | 10 | 320 | 320 | 10 |
|  |  |  | Day 21 - Serum | 163840 | 160 | 10 | 81920 | 320 | 10 |
|  |  |  | Day 42 - Serum | 5242880 | 2560 | 1205.2 | 5242880 | 2560 | 1525.691 |
|  |  |  | Day 56 - Serum | 2621440 | 2560 | 776.47 | 2621440 | 1280 | 711.395 |
| GP75 | Bivalent Vaccine | 50 µg | Day 0 - Serum | 320 | 160 | 10 | 160 | 320 | 10 |
|  |  |  | Day 21 - Serum | 40960 | 160 | 10 | 81920 | 640 | 10 |
|  |  |  | Day 42 - Serum | 1310720 | 1280 | 298.342 | 5242880 | 2560 | 706.733 |
|  |  |  | Day 56 - Serum | 655360 | 1280 | 158.984 | 2621440 | 2560 | 548.903 |
| GP76 | Bivalent Vaccine | 100 µg | Day 0 - Serum | 640 | 160 | 10 | 640 | 320 | 10 |
|  |  |  | Day 21 - Serum | 10240 | 160 | 10 | 20480 | 320 | 10 |
|  |  |  | Day 42 - Serum | 163840 | 320 | 57.412 | 1310720 | 640 | 206.433 |
|  |  |  | Day 56 - Serum | 81920 | 640 | 14.319 | 655360 | 2560 | 146.412 |
| GP77 | Bivalent Vaccine | 100 µg | Day 0 - Serum | 320 | 80 | 10 | 160 | 160 | 10 |
|  |  |  | Day 21 - Serum | 10240 | 160 | 10 | 10240 | 320 | 10 |
|  |  |  | Day 42 - Serum | 163840 | 160 | 50.72 | 327680 | 320 | 80.009 |
|  |  |  | Day 56 - Serum | 81920 | 160 | 12.993 | 327680 | 640 | 55.606 |
| GP78 | Bivalent Vaccine | 100 µg | Day 0 - Serum | 160 | 80 | 10 | 160 | 160 | 10 |
|  |  |  | Day 21 - Serum | 40960 | 160 | 10 | 40960 | 640 | 10 |
|  |  |  | Day 42 - Serum | 327680 | 320 | 123.549 | 1310720 | 2560 | 302.229 |
|  |  |  | Day 56 - Serum | 327680 | 320 | 81.135 | 655360 | 1280 | 170.475 |
| GP79 | Bivalent Vaccine | 100 µg | Day 0 - Serum | 320 | 160 | 10 | 160 | 160 | 1 |
|  |  |  | Day 21 - Serum | 163840 | 320 | 10 | 20480 | 640 | 10 |
|  |  |  | Day 42 - Serum | 1310720 | 1280 | 334.285 | 655360 | 640 | 183.019 |
|  |  |  | Day 56 - Serum | 655360 | 640 | 249.772 | 655360 | 640 | 137.632 |
