# Supplementary material for: Intranasal delivery of a bivalent norovirus vaccine formulated in an in situ gelling dry powder
Source: PLoS One. 2017 May 18;12(5):e0177310. doi: 10.1371/journal.pone.0177310 (PMC5436670; doi:10.1371/journal.pone.0177310)
Supplement: S3 Table — (DOCX) [file pone.0177310.s007.docx]

| Sample | | Antigen | | | Antigen Dose | | Time Point | G1 VLP | | GII.4 VLP | |
| --- | --- | --- | --- | --- | --- | --- | --- | --- | --- | --- | --- |
|  |  |  |  |  |  |  |  | IgG1 Titer | IgG2 Titer | IgG1 Titer | IgG2 Titer |
|  |  |  |  |  |  |  |  |  |  |  |  |
|  |  |  |  |  |  |  |  |  |  |  |  |
| 0 µg Dose Pooled Serum | | Bivalent Vaccine | | | 0 µg | | Day 0 | 20 | 20 | 20 | 20 |
|  |  |  |  |  |  |  | Day 21 | 20 | 20 | 20 | 20 |
|  |  |  |  |  |  |  | Day 42 | 20 | 20 | 20 | 20 |
|  |  |  |  |  |  |  | Day 56 | 20 | 40 | 20 | 20 |
| 5 µg Dose Pooled Serum | | Bivalent Vaccine | | | 5 µg | | Day 0 | 20 | 20 | 20 | 20 |
|  |  |  |  |  |  |  | Day 21 | 20 | 80 | 40 | 320 |
|  |  |  |  |  |  |  | Day 42 | 640 | 1280 | 640 | 20480 |
|  |  |  |  |  |  |  | Day 56 | 640 | 1280 | 1280 | 40960 |
| 15 µg Dose Pooled Serum | | Bivalent Vaccine | | | 15 µg | | Day 0 | 20 | 20 | 20 | 20 |
|  |  |  |  |  |  |  | Day 21 | 20 | 1280 | 40 | 1280 |
|  |  |  |  |  |  |  | Day 42 | 1280 | 10240 | 2560 | 81920 |
|  |  |  |  |  |  |  | Day 56 | 2560 | 20480 | 1280 | 81920 |
| 50 µg Dose Pooled Serum | | Bivalent Vaccine | | | 50 µg | | Day 0 | 20 | 20 | 20 | 20 |
|  |  |  |  |  |  |  | Day 21 | 320 | 10240 | 160 | 5120 |
|  |  |  |  |  |  |  | Day 42 | 20480 | 81920 | 10240 | 163840 |
|  |  |  |  |  |  |  | Day 56 | 20480 | 81920 | 20480 | 163840 |
| 100 µg Dose Pooled Serum | | Bivalent Vaccine | | | 100 µg | | Day 0 | 20 | 20 | 20 | 20 |
|  |  |  |  |  |  |  | Day 21 | 40 | 10240 | 80 | 2560 |
|  |  |  |  |  |  |  | Day 42 | 10240 | 20480 | 2560 | 81920 |
|  |  |  |  |  |  |  | Day 56 | 2560 | 40960 | 20480 | 81920 |
| Monovalent GI Pooled Serum | | GI Vaccine | | | 50 µg | | Day 0 | 20 | 20 | 20 | 40 |
|  |  |  |  |  |  |  | Day 21 | 320 | 20480 | 20 | 20 |
|  |  |  |  |  |  |  | Day 42 | 10240 | 81920 | 20 | 40 |
|  |  |  |  |  |  |  | Day 56 | 5120 | 40960 | 20 | 160 |
| Monovalent GII Pooled Serum | | GII.4 Vaccine | | | 50 µg | | Day 0 | 20 | 20 | 20 | 20 |
|  |  |  |  |  |  |  | Day 21 | 20 | 20 | 160 | 2560 |
|  |  |  |  |  |  |  | Day 42 | 20 | 640 | 10240 | 81920 |
|  |  |  |  |  |  |  | Day 56 | 20 | 160 | 5120 | 40960 |
|  |  | |  |  | |  |  |  |  |  |  |
