# Supplementary material for: Intranasal delivery of a bivalent norovirus vaccine formulated in an in situ gelling dry powder
Source: PLoS One. 2017 May 18;12(5):e0177310. doi: 10.1371/journal.pone.0177310 (PMC5436670; doi:10.1371/journal.pone.0177310)
Supplement: S5 Table — (DOCX) [file pone.0177310.s009.docx]

| Animal ID | Antigen | Antigen Dose | Sample Point | GI VLP | GII.4 VLP |
| --- | --- | --- | --- | --- | --- |
|  |  |  |  | IgG Titer | IgG Titer |
|  |  |  |  |  |  |
|  |  |  |  |  |  |
| GP52 | Bivalent Vaccine | 0 µg | Day 0 - Vaginal Lavage | 20 | 20 |
|  |  |  | Day 21 - Vaginal Lavage | 20 | 20 |
|  |  |  | Day 42 - Vaginal Lavage | 20 | 20 |
|  |  |  | Day 56 - Vaginal Lavage | 20 | 20 |
| GP53 | Bivalent Vaccine | 0 µg | Day 0 - Vaginal Lavage | 20 | 20 |
|  |  |  | Day 21 - Vaginal Lavage | 20 | 20 |
|  |  |  | Day 42 - Vaginal Lavage | 20 | 20 |
|  |  |  | Day 56 - Vaginal Lavage | 20 | 20 |
| GP54 | Bivalent Vaccine | 0 µg | Day 0 - Vaginal Lavage | 20 | 20 |
|  |  |  | Day 21 - Vaginal Lavage | 20 | 20 |
|  |  |  | Day 42 - Vaginal Lavage | 20 | 20 |
|  |  |  | Day 56 - Vaginal Lavage | 20 | 20 |
| GP55 | Bivalent Vaccine | 0 µg | Day 0 - Vaginal Lavage | 20 | 20 |
|  |  |  | Day 21 - Vaginal Lavage | 20 | 20 |
|  |  |  | Day 42 - Vaginal Lavage | 20 | 20 |
|  |  |  | Day 56 - Vaginal Lavage | 20 | 20 |
| GP56 | GI Vaccine | 50 µg | Day 0 - Vaginal Lavage | 20 | 20 |
|  |  |  | Day 21 - Vaginal Lavage | 640 | 20 |
|  |  |  | Day 42 - Vaginal Lavage | 1280 | 20 |
|  |  |  | Day 56 - Vaginal Lavage | 1280 | 20 |
| GP57 | GI Vaccine | 50 µg | Day 0 - Vaginal Lavage | 20 | 20 |
|  |  |  | Day 21 - Vaginal Lavage | 20 | 20 |
|  |  |  | Day 42 - Vaginal Lavage | 5120 | 20 |
|  |  |  | Day 56 - Vaginal Lavage | 640 | 20 |
| GP58 | GI Vaccine | 50 µg | Day 0 - Vaginal Lavage | 20 | 20 |
|  |  |  | Day 21 - Vaginal Lavage | 40 | 20 |
|  |  |  | Day 42 - Vaginal Lavage | 640 | 20 |
|  |  |  | Day 56 - Vaginal Lavage | 320 | 20 |
| GP59 | GI Vaccine | 50 µg | Day 0 - Vaginal Lavage | 20 | 20 |
|  |  |  | Day 2- Vaginal Lavage | 40 | 20 |
|  |  |  | Day 42 - Vaginal Lavage | 640 | 20 |
|  |  |  | Day 56 - Vaginal Lavage | 640 | 20 |
| GP60 | GII Vaccine | 50 µg | Day 0 - Vaginal Lavage | 20 | 20 |
|  |  |  | Day 21 - Vaginal Lavage | 20 | 2560 |
|  |  |  | Day 42 - Vaginal Lavage | 20 | 2560 |
|  |  |  | Day 56 - Vaginal Lavage | 640 | 40960 |
| GP61 | GII Vaccine | 50 µg | Day 0 - Vaginal Lavage | 20 | 20 |
|  |  |  | Day 21 - Vaginal Lavage | 20 | 40 |
|  |  |  | Day 42 - Vaginal Lavage | 20 | 320 |
|  |  |  | Day 56 - Vaginal Lavage | 20 | 640 |
| GP62 | GII Vaccine | 50 µg | Day 0 - Vaginal Lavage | 20 | 20 |
|  |  |  | Day 21 - Vaginal Lavage | 20 | 20 |
|  |  |  | Day 42 - Vaginal Lavage | 20 | 80 |
|  |  |  | Day 56 - Vaginal Lavage | 20 | 2560 |
| GP63 | GII Vaccine | 50 µg | Day 0 - Vaginal Lavage | 20 | 20 |
|  |  |  | Day 21 - Vaginal Lavage | 20 | 20 |
|  |  |  | Day 42 - Vaginal Lavage | 20 | 80 |
|  |  |  | Day 56 - Vaginal Lavage | 20 | 20 |
| GP64 | Bivalent Vaccine | 5 µg | Day 0 - Vaginal Lavage | 20 | 20 |
|  |  |  | Day 21 - Vaginal Lavage | 20 | 20 |
|  |  |  | Day 42 - Vaginal Lavage | 20 | 160 |
|  |  |  | Day 56 - Vaginal Lavage | 20 | 80 |
| GP65 | Bivalent Vaccine | 5 µg | Day 0 - Vaginal Lavage | 20 | 20 |
|  |  |  | Day 21 - Vaginal Lavage | 20 | 20 |
|  |  |  | Day 42 - Vaginal Lavage | 20 | 20 |
|  |  |  | Day 56 - Vaginal Lavage | 20 | 20 |
| GP66 | Bivalent Vaccine | 5 µg | Day 0 - Vaginal Lavage | 20 | 20 |
|  |  |  | Day 21 - Vaginal Lavage | 20 | 40 |
|  |  |  | Day 42 - Vaginal Lavage | 20 | 160 |
|  |  |  | Day 56 - Vaginal Lavage | 320 | 2560 |
| GP67 | Bivalent Vaccine | 5 µg | Day 0 - Vaginal Lavage | 20 | 20 |
|  |  |  | Day 21 - Vaginal Lavage | 20 | 20 |
|  |  |  | Day 42 - Vaginal Lavage | 80 | 1280 |
|  |  |  | Day 56 - Vaginal Lavage | 160 | 1280 |
| GP68 | Bivalent Vaccine | 15 µg | Day 0 - Vaginal Lavage | 20 | 20 |
|  |  |  | Day 21 - Vaginal Lavage | 20 | 20 |
|  |  |  | Day 42 - Vaginal Lavage | 20 | 320 |
|  |  |  | Day 56 - Vaginal Lavage | 20 | 160 |
| GP69 | Bivalent Vaccine | 15 µg | Day 0 - Vaginal Lavage | 20 | 20 |
|  |  |  | Day 21 - Vaginal Lavage | 20 | 20 |
|  |  |  | Day 42 - Vaginal Lavage | 320 | 1280 |
|  |  |  | Day 56 - Vaginal Lavage | 320 | 1280 |
| GP70 | Bivalent Vaccine | 15 µg | Day 0 - Vaginal Lavage | 20 | 20 |
|  |  |  | Day 21 - Vaginal Lavage | 20 | 40 |
|  |  |  | Day 42 - Vaginal Lavage | 80 | 320 |
|  |  |  | Day 56 - Vaginal Lavage | 80 | 320 |
| GP71 | Bivalent Vaccine | 15 µg | Day 0 - Vaginal Lavage | 20 | 20 |
|  |  |  | Day 21 - Vaginal Lavage | 20 | 40 |
|  |  |  | Day 42 - Vaginal Lavage | 160 | 160 |
|  |  |  | Day 56 - Vaginal Lavage | 40 | 40 |
| GP72 | Bivalent Vaccine | 50 µg | Day 0 - Vaginal Lavage | 20 | 20 |
|  |  |  | Day 21 - Vaginal Lavage | 20 | 20 |
|  |  |  | Day 42 - Vaginal Lavage | 320 | 640 |
|  |  |  | Day 56 - Vaginal Lavage | 80 | 160 |
| GP73 | Bivalent Vaccine | 50 µg | Day 0 - Vaginal Lavage | 20 | 20 |
|  |  |  | Day 21 - Vaginal Lavage | 20 | 20 |
|  |  |  | Day 42 - Vaginal Lavage | 40 | 40 |
|  |  |  | Day 56 - Vaginal Lavage | 20 | 20 |
| GP74 | Bivalent Vaccine | 50 µg | Day 0 - Vaginal Lavage | 20 | 20 |
|  |  |  | Day 21 - Vaginal Lavage | 1280 | 640 |
|  |  |  | Day 42 - Vaginal Lavage | 20480 | 20480 |
|  |  |  | Day 56 - Vaginal Lavage | 5120 | 5120 |
| GP75 | Bivalent Vaccine | 50 µg | Day 0 - Vaginal Lavage | 20 | 20 |
|  |  |  | Day 21 - Vaginal Lavage | 320 | 640 |
|  |  |  | Day 42 - Vaginal Lavage | 1280 | 5120 |
|  |  |  | Day 56 - Vaginal Lavage | 2560 | 5120 |
| GP76 | Bivalent Vaccine | 100 µg | Day 0 - Vaginal Lavage | 20 | 20 |
|  |  |  | Day 21 - Vaginal Lavage | 20 | 40 |
|  |  |  | Day 42 - Vaginal Lavage | 160 | 640 |
|  |  |  | Day 56 - Vaginal Lavage | 40 | 320 |
| GP77 | Bivalent Vaccine | 100 µg | Day 0 - Vaginal Lavage | 20 | 20 |
|  |  |  | Day 21 - Vaginal Lavage | 20 | 20 |
|  |  |  | Day 42 - Vaginal Lavage | 80 | 320 |
|  |  |  | Day 56 - Vaginal Lavage | 40 | 160 |
| GP78 | Bivalent Vaccine | 100 µg | Day 0 - Vaginal Lavage | 20 | 20 |
|  |  |  | Day 21 - Vaginal Lavage | 40 | 80 |
|  |  |  | Day 42 - Vaginal Lavage | 640 | 1280 |
|  |  |  | Day 56 - Vaginal Lavage | 640 | 2560 |
| GP79 | Bivalent Vaccine | 100 µg | Day 0 - Vaginal Lavage | 20 | 20 |
|  |  |  | Day 21 - Vaginal Lavage | 1280 | 320 |
|  |  |  | Day 42 - Vaginal Lavage | 1280 | 1280 |
|  |  |  | Day 56 - Vaginal Lavage | 640 | 320 |
